# A multicenter case control study of association of vitamin D with breast cancer among women in Karachi, Pakistan

**DOI:** 10.1371/journal.pone.0225402

**Published:** 2020-01-22

**Authors:** Uzma Shamsi, Shaista Khan, Iqbal Azam, Aysha Habib Khan, Amir Maqbool, Mohammad Hanif, Tiffany Gill, Romaina Iqbal, David Callen

**Affiliations:** 1 School of Medicine, University of Adelaide, Adelaide, Australia; 2 Department of Surgery, Aga Khan University, Karachi, Pakistan; 3 Department of Community Health Sciences, Aga Khan University, Karachi, Pakistan; 4 Department of Pathology & Laboratory Medicine, Aga Khan University, Karachi, Pakistan; 5 Department of Oncology, Karachi Institute of Radiation and Nuclear Medicine Hospital (KIRAN), Karachi, Pakistan; University of Extremadura, SPAIN

## Abstract

**Background:**

The prevalence of vitamin D inadequacy and breast cancer are both high among women living in Karachi, Pakistan.

**Methods:**

A matched case control study was conducted in two hospitals of Karachi, Pakistan to evaluate the association of vitamin D (serum 25-hydroxyvitamin D) concentrations, vitamin D supplementation and sun exposure with breast cancer among Pakistani women. A total of 411 newly diagnosed histologically confirmed primary breast cancer cases were enrolled and 784 controls, free of breast and any other cancers, were matched by age (year of birth ± 5 years), residence in the same geographic area and study site. Information was collected on sociodemographic history, history of vitamin D supplementation, past medical and obstetrical history, family history of breast cancer, sun exposure history, histopathology reports and anthropometric measurement and venous blood was collected to measure serum 25-hydroxyvitamin D (25(OH)D) concentration.

**Results:**

Compared to patients with sufficient serum vitamin D (>30 ng/ml), women with serum vitamin D deficiency (<20ng/ml), had a higher risk of breast cancer (OR = 1.65, 95%CI: 1.10, 2.50). Women with history of vitamin D supplementation one year prior to enrollment, had significant protective effect against breast cancer (OR = 0.32, 95% CI: 0.24, 0.43).

**Conclusions and recommendation:**

Serum vitamin D deficiency was associated with increased risk of breast cancer, while vitamin D supplementation was associated with decreased risk of breast cancer. In Pakistani women, where vitamin D deficiency is common, raising and maintaining serum vitamin D at population level is a safe and affordable strategy. It may play a role in reducing the incidence of both vitamin D deficiency and breast cancer, particularly among poor women where the breast cancer mortality is highest due to limited resources for early detection, diagnosis, and treatment. The effects of vitamin D with regard to breast cancer risk in Karachi Pakistan should be further evaluated.

## Background

Cancer rates and the financial burdens of cancer treatments are increasing worldwide in both developed and developing countries [1, 2]. In developing countries, where health systems are not sufficiently well resourced to provide the optimum facilities for cancer diagnosis and cancer management, cancer is particularly associated with high morbidity and mortality [3]. Expensive targeted cancer treatments continue to advance the treatment for breast cancer in more affluent countries. However, in developing economies, like Pakistan, the exploration of more affordable cancer prevention, and management is a necessity but currently there is limited cancer prevention research and available strategies [4].

There is substantial evidence supporting the theory that hormonal mechanisms play a vital role in the etiology of breast cancer. Epidemiological studies and laboratory findings indicate that vitamin D has potential anticancer activity [5]. An individual’s serum 25(OH)D concentrations are principally determined by the amount of the skin’s ultraviolet (UVB) radiation exposure from the sun and this can also be influenced by skin pigmentation. In addition, there are dietary sources of vitamin D, particularly oily fish, and easily affordable and accessible vitamin D supplements. In a meta-analyses of observational studies and randomized controlled trials by Theodoratou, et al, out of 137 diseases linked with vitamin D deficiency (including cardiovascular, autoimmune, infectious, skeletal, malignant, metabolic, and other diseases), ten outcomes were studied and results showed an association of vitamin D deficiency (VDD) with birth weight, childhood dental caries, and parathyroid hormone concentrations in patients suffering from chronic kidney disease [6]. In other studies, low 25(OH)D concentration (<20ng/ml) was associated with multiple types of adverse musculoskeletal diseases and non-musculoskeletal health problems, for example cardiovascular diseases [7], hypertension, type 2 diabetes mellitus [8] and cancers, osteomalacia, rickets, hypophosphatemia, osteoporosis, autoimmune diseases (e.g. psoriasis) and infectious diseases [9], and therefore, there are national programs that vary among countries to provide dietary vitamin D fortified foods [10]. It has also been well established that low concentrations of 25 (OH)D are associated with an increased risk of some cancers, for example colorectal cancer [11, 12]. In a recent meta-analysis, it was found that there was a highly significant dose-response relationship between serum 25(OH)D concentrations and overall survival in breast cancer patients [13].

Despite the biological plausibility that vitamin D has an anticancer role, the literature on the relationship between breast cancer and 25(OH)D concentrations remains more controversial. The research findings evaluating the role of vitamin D as a possible preventive agent against breast cancer vary, and the optimal serum 25(OH)D concentration for cancer and other diseases prevention is still unknown [14]. Overall, the results from a number of cell line experiments, mouse studies, ecological studies, observational studies and some clinical trials indicate that vitamin D has anticancer activity [15].

Ecological studies on large population living in diverse countries including Europe, China, Australia, France Japan and the USA have also consistently shown the protective association of increased UVB sun exposure and latitude on reducing breast cancer incidence and mortality [16–19]. However, ecologic fallacy and lack of data relating to serum 25(OH)D concentrations at an individual level are major weaknesses of such studies.

Results from observational studies show a protective association of increased 25(OH)D concentrations against breast cancer which is observed more frequently in case control studies than in cohort studies [20–26]. Prospective studies investigating the role of vitamin D in breast cancer have mixed results [27–31] with some studies showing no association with breast cancer [31–37]. A case–control study nested within the Nurses’ Health Study II (NHSII) did not show any association of breast cancer with vitamin D [30]. In addition, in a cohort study from 1965–1976, it was found that there was no difference in the pre diagnostic concentrations of 1,25(OH)_2_D between cases of breast cancer and their matched control subjects [29]. These inconsistent results of association of serum vitamin D with breast cancer can be due to multiple factors like different population, and tumor biology. One problem with cohort studies is that there is sometimes a long period of time between the blood draw for vitamin D and the outcome observed. That long-time gap may explain some of the discrepancy between the findings of case-control studies and cohort studies.

There is dearth of well-designed and conducted large randomized controlled trials (RCTs) to assess if vitamin D supplementation can increase serum 25(OH)D concentrations and reduce the incidence of breast cancer. Results vary, with some clinical trials supporting the results of association studies [38, 39], while others showing an absence of cancer prevention by vitamin D [40]. Some of the limitations of RCTs done are inadequate sample sizes and limited time of follow-up. There have been several meta-analyses of case-control studies that demonstrate an association of high serum 25(OH)D with lower incidence of breast cancer [41–44]. A meta-analysis of prospective studies showed a 12% protective association of serum 25(OH)D of 27-<35 ng/mL with breast cancer, but there was no more protective effects above 35 ng/ml in a dose-response analysis [45]. In another meta-analysis by Hong et al, a serum concentration of greater than 30 ng/ml of serum 25(OH)D was associated with decreased risk of breast cancer [46]. The protective association has also been observed in postmenopausal but not in premenopausal women in another meta-analysis by Stearns V et al. [47]. A meta-analysis of 14 studies carried out by Wang et al, that included 9110 breast cancer cases and 16,244 controls showed an overall protective effect of high serum 25(OH)D on breast cancer risk (RR = 0.84, 95% CI = 0.750–0.951) [48] with a 3.2% reduction of breast cancer risk with every 10 ng/ml increase in serum 25(OH)D concentrations (p<0.001) in postmenopausal women only [48]. However, there was no protective effect of 25(OH)D on breast cancer risk among premenopausal women [48]. Similarly, a meta- analysis of 6 studies suggested that higher intakes of vitamin D were associated with a lower risk of breast cancer [49]. A recent review based on cohort studies only did not find any association of vitamin D with breast cancer but it excluded consideration of cross-sectional, case control studies and ecological studies [50]. On the contrary, in two cohort studies: ESTHER (Germany) and TROMSO (Norway); and a subset nested-case control study from EPIC-Elderly (Greece, Denmark, Netherlands, Spain and Sweden) consisting of a total of 15,486 individuals, there was some evidence of increased breast cancer with higher pre-diagnostic 25(OH)D concentrations [51]. Overall the meta-analyses do provide evidence of a chemo-preventive role of vitamin D against breast cancer but there is a need of further randomized control trials, particularly in populations with extremes of serum 25(OH)D concentrations.

Studies in Asian population of breast cancer and the possible relationship with 25(OH)D concentrations, are limited [52–54]. A study from Jordon, although with limited number of women, did show an association of breast cancer with VDD [55]. Results from a case-control study in Iran also support the protective effect of higher serum concentration of 25(OH)D against breast cancer [56]. Another case control study in Asian women also suggests potential preventive effect of vitamin D on breast cancer for Korean women [57]. Similar findings of protective role of vitamin D against breast cancer are reported from studies in Indian populations [52, 58]. Two studies done in Pakistan have only descriptive analysis in one [59] and small sample size in the other study [60].

Since Pakistani population was reported to have low concentrations of serum 25(OH)D [61] and high rates of breast cancer [62], we conducted a case control study in this population examining the association between women’s serum 25(OH)D concentrations and breast cancer risk. In addition, the major sources of vitamin D, endogenous cutaneous synthesis through UVB sun exposure and exogenous through intake of vitamin D supplements, were evaluated in this population. Serum 25(OH)D concentrations of women were determined since this is the best biomarker to measure vitamin D status [63].

## Methods

### Study design and setting

This matched case control study was conducted in Karachi, the 5th most populous city in the world with an estimated population of 24 million in 2017. Study participants were sourced from Aga Khan University Hospital (AKUH), which is a private JCIA accredited academic medical center, and the Karachi Institute of Radiation and Nuclear Medicine Hospital (KIRAN) which is a leading referral and cancer treatment public hospital and provides financial assistance for diagnosis and treatment on welfare basis, to more than 75% of its patients, who come from all over Pakistan.

### Study participants

All women who were newly diagnosed with a primary histologically confirmed case of breast cancer between February 2015 to July 2018 were eligible as cases. Women who were extremely sick, unable to complete the interview, been living outside Pakistan for more than a year and diagnosis of breast cancer for greater than 6 months before study enrolment were excluded. Women receiving adjuvant or neoadjuvant chemotherapy or radiation, with diagnosis of non-epithelial breast tumor (ICD-O histology code 8800), and women lost to follow up during their metastatic and laboratory work up were also excluded from the study.

For each patient two controls free of any cancer diagnosis were matched by age (year of birth ± 5 years), residence in the same geographic area and hospital. The control women were recruited from those attending in- and out-patient services for general medical, and surgical departments of the two participating hospitals and who had no previous diagnosis of breast cancer or any other cancer. The controls enrolled from surgery were those who came for follow up of mammography screening, with benign breast symptoms. Controls sourced from general medicine clinics had a range of medical conditions, for example diabetes, hypertension, headaches and anxiety. All efforts were made to avoid selection bias by proper selection of cases and controls and standardized, and uniform data collection procedures.

The number of enrolled cases and controls subjects was decided based on certain assumptions. We assumed the prevalence of deficiency of vitamin D and other risk factors amongst the control group to be in the range of 10–90%. In an audit of blood samples at Aga Khan University, VDD noted was 66.1% [64]. In order to be able to detect an odds ratio of at least 2 with a power of 80%, at a significance concentration of 5% and considering a 1: 2 ratio between cases and controls, we calculated a sample size of 400 cases and 800 controls. However, in the final achieved sample, a total of 411 breast cancer cases and 784 control subjects were enrolled (55 stratas had 1:1 case control ratio).

### Ethics approval and consent to participate

The ethical approval was obtained by the Human Research Ethics Committee of the University of Adelaide and the Ethical Review Committees of two hospitals in Karachi Pakistan: Aga Khan University Hospital AKUH & Karachi Institute of Radiation and Nuclear Medicine Hospital KIRAN. Patients who were literate read and signed the informed consent form, and verbal consent was obtained from those who could not read or write.

### The pilot study and pretesting

In the first phase, a pilot study was undertaken to pretest the complete questionnaire on a sample of 50 patients attending the outpatient department of Jinah Postgraduate Medical Center JPMC and AKUH between Aug 2014-Jan 2015. Those subjects were not included in the main study. The questionnaire was revised and finalized based on the pretest results, to ensure standardization and reliability of the questionnaire in urdu, the local language.

### Questionnaire

All consenting participants were interviewed using the same structured questionnaire. The questionnaire was divided into different sections of sociodemographic history, history of any vitamin D supplementation, and past medical and obstetrical history, family history of breast cancer or any other cancer, sun exposure history, histopathology reports and anthropometric measurement. Where relevant, clinical notes and pathology reports were assessed. Socio demographic factors included age, ethnicity, education, marital status, place and type of residence, number of dependants and current employment status. Education was treated as an ordinal variable and categorized as <8, 8–12, or > 12 years of full-time study. Socio-economic (SES) factors included education, place and type of residence, crowding index, home ownership, number of rooms, total household members and total household monthly income. Crowding index was also calculated as number of household members divided by number of rooms and was further categorized as <1, 1–2, >2. Factor analysis was used to identify the important variables for socioeconomic status and a composite variable was calculated for socioeconomic status and categorized into upper, middle and poor.

Participants self-reported their family history of breast cancer as none (no first-degree family history), or one first degree relative and age at diagnosis of breast cancer, or multiple affected first-degree relatives or second-degree relatives and ages at breast cancer diagnosis. Other variables assessed included reproductive history, body mass index (BMI), and history of benign breast disease. Histopathology and estrogen receptor (ER), progesterone receptor (PR) and human epidermal growth factor receptor (HER2/ neu) status of breast cancer cases were retrieved from medical records. Tumor characteristics relating to tumor size (T), nodes involved (N), and presence of metastasis (M) were also extracted from pathology reports and cancer staging expressed as stage I (the least advanced stage) to stage IV (the most advanced stage).

The STROBE standardised reporting guidelines for case control studies were followed to ensure standardised conduct and reporting of the study [65].

#### Measurement of serum 25(OH)D concentrations

For all enrolled subjects venous blood samples (2.5 ml) were collected in yellow topped gel tubes. For breast cancer patients, collection was within 30 days of diagnosis of cancer and prior to chemotherapy, and from controls, at the end of the questionnaire interview. The collected blood was transported on the same day to the Section of Clinical Chemistry, Department of Pathology & Laboratory Medicine, at AKUH for biochemical analysis. Serum was separated by centrifugation and stored at -80°C until analysis. Serum 25(OH)D concentrations was measured by a kit from DiaMetra S.r.I. Headquarter: Via Garibaldi, 18–20090 SEGRATE (MI) Italy, using solid phase enzyme-linked immunoassay (ELISA) based on the principle of competitive binding. A dose response curve was used to ascertain 25(OH)D concentrations range in unknown specimens by using five human serum-based calibrators from C0 to C5 with concentration range from 0–120 ng/ml. The lowest detectable concentration of 25(OH)D that can be distinguished from the calibrator 0 was 0.3 ng/ml at 95% confidence limit. The assay had intra assay variability and inter assay variability of < 6.4% and <6.955 respectively. Five low and high vitamin D control samples provided with the kit were run with each batch of samples analysed for quality control. The AKUH laboratory participates in the College of American Pathologists CAP proficiency testing. The cut-off used to define VDD is the point where parathyroid hormone (PTH) starts to rise [66]. Vitamin D deficient status was defined as serum 25(OH)D concentration of 12–19 ng/ml, vitamin D insufficient as 20-30ng/ml and vitamin D sufficient as >30ng/ml.

#### Vitamin D supplements intake

The use of vitamin D supplement (both injections and oral), was assessed in relation to the index year, defined as one year prior to breast cancer diagnosis for cases or the interview for controls. Vitamin D supplementation was assessed by asking participants if they had been taking vitamin D supplements regularly, occasionally or not at all in their lifetime, with the mode of administration identified as either injections, oral tablets or ampoules. Similar information was used to assess use of multivitamins, name of the multivitamins, and vitamin D calcium combination supplementation with details about frequency of intake and the brands of tablets (where available) were also determined.

#### Sun exposure measurement questionnaire

The geographic location of Karachi (latitude 24.51’N, longitude 67°02′E and elevation 8 metres) served as a surrogate measure for ultraviolet B (UVB) exposure. It should be noted that the climate of Karachi provides ample sunlight throughout the year for maintaining adequate 25(OH)D concentrations. To assess sun exposure among cases and controls, the validated long-term sun exposure measurement questionnaire (LT SEM-Q) was used. This questionnaire has been validated against gold standard ultraviolet (UV) dosimeters [67]. This questionnaire estimates retrospective sunlight exposure throughout a patient’s lifetime, which is especially relevant for chronic diseases like breast cancer that develop over many years. The questionnaire assessed duration of sun exposure to various parts of the body, skin tone of the individual, use of sunscreens and other cosmetics, and sun avoidance behaviour. The time spent outdoors between 10 am and 4 pm in summers and winters was assessed to estimate the amount of time in minutes per day and per week each participant was exposed to UVB radiations. Other factors such as atmospheric pollution, latitude, and season depend on the urban or rural setting and province of the enrolled subjects were addressed by matching cases and controls on region of residence.

Skin tone of the participants was assessed against a shade card by matching shade of the skin on the inner side of the forearm (unexposed part) of the participant and forehead (exposed part) with the shade on the card, according to LT SEM-Q, to match the skin tones of Asian population. Details of weights given to sun exposure variables are shown in S1 Table. The final scoring algorithm of sun exposure score in summers and winters was created by multiplying the time (minutes) spent in the sun by the proportions of the different variables.

## Statistical analysis

Conditional logistic regression with matched sets as strata was used to compute odds ratios (ORs) and 95% confidence intervals (CIs) to evaluate the association of 25(OH)D, sun exposure, and vitamin D supplements with breast cancer. We examined potential confounding by the following covariates as categorical variables: socioeconomic status, education, parity, body mass index (BMI), first-degree family history of breast cancer, breastfeeding, and menopausal status. SPSS 22.0 software (IBM Statistics, Armonk, New York, USA) was used where a p-value< 0.05 was accepted as significant for all statistical tests.

## Results

Table 1 presents the median, minimum, maximum and mean with SD for continuous data of serum 25(OH)D concentrations showing high prevalence of VDD among both cases and controls. Individual 25(OH)D concentrations encompassed a broad range from highly deficient (0.3 ng/ml) to exceptionally high (165.5 ng/ml). The median 25(OH)D concentrations among breast cancer cases was lower (15.3 ng/ml) compared to controls (16.7 ng/ml).

**Table 1:** Serum concentration of vitamin D among cases and controls.

Serum vitamin D (ng/ml)	Median (SD)	Minimum	Maximum	Mean	SD
**Cases**	15.3 (15.6)	0.3	165.5	20.1	21.3
**Controls**	16.7 (20.1)	0.9	149.0	23.0	20.3

To enable a more rigorous statistical analysis, cases and controls were categorized into four different concentrations of 25(OH)D, defined as severely deficient (<12 ng/ml), deficient (12–19 ng/ml), insufficient (20-30ng/ml) and sufficient (>30ng/ml) (Table 2). VDD was significantly more frequent in breast cancer cases, with 38.9% of breast cancer cases categorized as severe VDD compared to 32.9% of controls, while 28.8% of breast cancer cases were classified as deficient compared to 23.8% of controls. Vitamin D sufficiency was higher among controls (24.8%) compared to cases (17.4%).

**Table 2:** Number and percentage of breast cancer cases and controls for different serum 25(OH)D concentrations.

Serum 25(OH)D (ng/ml)		Case		Control		All participants		p value*
		n	%	n	%	n	%	
								0.02
**Severely deficient**	**<12**	112	38.9	202	32.9	314	34.8	
**Deficient**	**12–19**	83	28.8	146	23.8	229	25.4	
**Insufficient**	**20–30**	43	14.9	114	18.6	157	17.4	
**Sufficient**	**>30**	50	17.4	152	24.8	202	22.4	

*p values generated from Chi-square or Fisher Exact test

Overall 60.2% of women in Karachi had serum concentrations of 25(OH)D < 20ng/ml, which is considered to indicate vitamin D deficiency. A severe deficiency of serum 25(OH)D <12 ng/ml was reported among 34.8% of women.

Table 3 presents the demographic and reproductive characteristics of all the study participants stratified according to serum 25(OH)D concentrations, compared by chi-square tests. Note the severely deficient and deficient 25(OH)D categories were combined in this and subsequent analyses. Vitamin D deficient women, compared with vitamin D sufficient women, were significantly more likely to be in the 35-44-year age group, premenopausal, and of lower SES.

**Table 3:** Sociodemographic, reproductive and anthropometric characteristics of women stratified according to serum 25(OH)D concentrations.

Variable	category	Serum 25(OH)D concentration (ng/ml)						
		Deficient (<20)		Insufficient (20–30)		Sufficient (>30)		p value*
		n = 543		n = 157		n = 202		
		n	%	n	%	n	%	
**Age groups (years)**								<0.001
	**<35**	100	18.4	19	12.1	22	10.9	
	**35–45**	209	38.5	55	35.0	54	26.7	
	**46–54**	128	23.6	36	22.9	52	25.7	
	**55 & above**	106	19.5	47	29.9	74	36.6	
**Education**								0.57
	**<grade 8**	140	25.8	31	19.7	49	24.3	
	**grades 8–12**	178	32.8	56	35.7	63	31.2	
	**>grade 12**	224	41.3	70	44.6	90	44.6	
**Socioeconomic status (SES)**								0.002
	**upper**	62	11.4	34	21.7	35	17.3	
	**middle**	308	56.7	84	53.5	122	60.4	
	**lower**	173	31.9	39	24.8	45	22.3	
**Parity**								0.02
	**nullipara**	85	15.7	14	8.9	30	14.9	
	**≤3**	232	42.7	85	54.1	105	52.0	
	**>3**	226	41.6	58	36.9	67	33.1	
**Breastfeeding**								0.11
	**no**	12	2.6	9	6.3	7	4.1	
	**yes**	443	97.4	133	93.7	164	95.9	
**Menopause**								<0.001
	**menopause**	247	45.7	83	53.2	123	61.8	
	**pre menopause**	293	54.3	73	46.8	76	38.2	
**Family history of breast cancer**								0.46
	**yes**	123	22.7	40	25.5	54	26.7	
	**no**	420	77.3	117	74.5	148	73.3	
Body mass index**								0.66
	**<23**	83	16.3	21	14.4	39	20.0	
	**23–25**	71	14.0	23	15.8	25	12.8	
	**>26**	354	69.7	102	69.9	131	67.2	

*p values generated from Chi-square or Fisher Exact test,

**Weight (kg)/height (m)2

Those women with VDD and insufficiency on average had higher parity. This is also likely to be related to their lower SES status.

Table 4 presents the distribution of tumor characteristics within the three different serum 25(OH)D concentrations. There was no significant association of 25(OH)D concentrations with any of these tumor characteristics.

**Table 4:** Association between serum 25(OH)D concentrations(ng/ml) and the tumor characteristics among breast cancer cases.

Variable	category	Deficient (<20)		Insufficient (20–30)		Sufficient (>30)		p value*
		n	%	n	%	n	%	
**Histopathology**	**IDC**	160	85.6	36	87.8	40	80.0	0.39
	**lobular**	7	3.7	1	2.4	5	10.0	
	**Others**	20	10.7	4	9.8	5	10.0	
**ER**	**positive**	111	67.7	19	63.3	32	74.4	0.57
	**negative**	53	32.3	11	36.7	11	25.6	
**PR**	**positive**	98	59.8	15	50.0	32	74.4	0.09
	**negative**	66	40.2	15	50.0	11	25.6	
**Grade of tumor**	**III**	74	41.6	13	35.1	11	23.9	0.08
	**I/II**	104	58.4	24	64.9	35	76.1	
**Tumor size**	**≤5cm**	98	55.4	20	57.1	24	55.8	0.98
	**>5cm**	79	44.6	15	42.9	19	44.2	
**Nodes**	**NoN1**	121	72.9	29	82.9	29	69.0	0.37
	**N2**	30	18.1	2	5.7	7	16.7	
	**N3**	15	9.0	4	11.4	6	14.3	
**Metastasis**	**no metastasis**	130	81.8	24	80.0	31	81.6	0.97
	**metastasis**	29	18.2	6	20.0	7	18.4	
**TNM Stage**	**Stage I**	23	15.5	10	34.5	5	13.9	0.24
	**Stage II**	53	35.8	8	27.6	11	30.6	
	**Stage III**	43	29.1	5	17.2	13	36.1	
	**Stage IV**	29	19.6	6	20.7	7	19.4	

*p values generated from Chi-square or Fisher Exact test

Abbreviations ER Estrogen receptor PR progesterone receptor

The use of different forms of vitamin D and multivitamin supplementation was assessed among cases and controls. For each form of supplementation, the breast cancer group had a significantly lower usage than the control group (Table 5).

**Table 5:** Association of different forms of vitamin D and multivitamin supplementation with breast cancer.

Vitamin D supplementation	category	Case (411)		Control (784)		OR 95% CI	p value*
		n	%	n	%		
**Vitamin D tablets**							<0.001
	**Yes**	27	6.7	154	19.6	0.28 (0.18–0.44)	
	**No**	375	93.3	630	80.4	Ref (1)	
**Oral vitamin D Drops**							<0.001
	**Yes**	15	3.7	67	8.5	0.42 (0.23–0.75)	
	**No**	387	96.3	717	91.5	Ref (1)	
**Vitamin D calcium tablets**							<0.001
	**Yes**	83	20.6	264	33.7	0.47 (0.35–0.64)	
	**No**	319	79.4	520	66.3	Ref (1)	
**Injection vitamin D**							<0.001
	**Yes**	69	17.2	254	32.4	0.41(0.3–0.56)	
	**No**	333	82.8	530	67.6	Ref (1)	
**Multivitamin**							<0.001
	**Yes**	100	24.9	300	38.3	0.52 (0.39–0.68)	
	**No**	302	75.1	484	61.7	Ref (1)	

*p values generated from Chi-square or Fisher Exact test

OR = odds ratio, CI = confidence interval

There was a significant association between serum 25(OH)D concentrations and intake of vitamin D supplements with 72% of breast cancer cases in the VDD group being non-users of vitamin D supplementation compared to 50.6% of controls. Vitamin D users had higher serum vitamin D sufficiency among both cases and controls (S2 Table).

There was no difference in the total sun exposure score between cases and controls and therefore no association with breast cancer risk (Table 6). However, when component variables of sun exposure score were analyzed separately, women with more sun avoidance behavior and an attire covering their head, neck, full legs and full arms had an increased risk of breast cancer. This was also associated with increased VDD (data not shown). Further detail of distribution of sun exposure score and its component variables among breast cancer cases and controls is presented in S3 Table.

**Table 6:** Association of sun exposure variables including body parts exposed and sun avoidance behavior with breast cancer.

	Variables		case		control		OR (95% (CI)	p value
			n	%	n	%		
**IA**	**Total sun exposure score (mean SD)** *							
	Score of Sun exposure in Summer per week		32.2	99.5	36.4	79.1	1.00 (1.01–1.02)	0.98
	Score of Sun exposure in Winter per week		56.4	97.6	48.0	117.6	1.00 (0.99–1.00)	0.90
**IB**	**Individual components of sun exposure score**							
	Head Covered	yes	332	82.4	572	73.0	1.97 (1.41–2.74)	<0.001
		no	71	17.6	210	27.0	1 (Ref)	
	Face Covered	yes	97	23.8	161	20.5	1.29 (0.94–1.76)	0.11
		no	306	76.2	621	79.5	1 (Ref)	
	Neck Covered	yes	255	63.3	426	54.5	1.53 (1.18–1.99)	0.001
		no	148	36.7	356	45.5	1 (Ref)	
	Full Arm Covered	yes	299	74.2	537	68.7	1.37 (1.03–1.82)	0.03
		no	104	25.8	245	31.3	1 (Ref)	
	Hands Covered	yes	11	2.7	14	1.8	1.78 (0.74–4.29)	0.19
		no	392	97.3	768	98.2	1 (Ref)	
	Full Legs Covered	yes	389	96.5	760	97.2	0.77 (0.35–1.66)	0.76
		no	14	3.5	22	2.8	1 (Ref)	
	Sun avoidance behavior	yes	361	89.6	660	84.4	1.73 (1.14–2.62)	0.01
		no	42	10.4	122	15.6	1 (Ref)	
	Skin Tone Forehead (mean SD) *		5.7	1.5	5.3	1.4	1.19 (1.08–1.31)	<0.001
	attire outside	chadder**	146	36.3	214	27.4	2.28 (1.62–3.22)	<0.001
		burqa***	177	44.0	320	41.0	1.89 (1.35–2.66)	
		other attires	79	19.7	247	31.6	1 (Ref)	

*Mean SD

** attire covering head, full arms and full legs

***attire covering head, face, neck, full arms and full legs

Table 7 presents multivariable analysis showing that women with serum VDD (<20ng/ml), had a higher risk of breast cancer compared to patients with sufficient serum vitamin D (>30 ng/ml) (OR = 1.65, 95%CI: 1.10, 2.50). Women with a history of vitamin D supplementation were significantly protected against breast cancer (OR = 0.32, 95% CI: 0.24, 0.43). Women wearing the chadder and/or burqa resulting in minimal exposure to sunlight had a higher breast cancer rate compared to women without those attires (OR = 1.80, 95% CI: 1.25, 2.59) and (OR = 1.50, 95% CI: 1.05, 2.16) respectively.

**Table 7:** Final multivariable adjusted ORs (95% CIs) of vitamin D with breast cancer among women in Karachi Pakistan (n = 1195).

Variable	Category	Crude OR (95% CI)	Adjusted OR (95% CI)
**Serum 25(OH)D concentration(ng/ml)**			
	**<20**	1.83 (1.25–2.67)	1.65 (1.10–2.50)
	**20–30**	1.56 (1.08–2.23)	1.17 (0.68–2.01)
	**>30**	1 (Ref)	1 (Ref)
**Use of vitamin D supplementation**	**yes**	0.28(0.18–0.44)	0.32 (0.24–0.43)
	**no**		1 (Ref)
**Attire outside**	chadder**	2.28(1.62–3.22)	1.80 (1.25–2.59)
	burqa***	1.89(1.34–2.66)	1.50 (1.05–2.16)
	**other attires**	1 (Ref)	1 (Ref)

Adjusted for socioeconomic status, education, parity, body mass index (BMI), first-degree family history of breast cancer, breastfeeding, and menopausal status.

** covering head, full arms and full legs

***covering head, face, neck, full arms and full legs

## Discussion

In this study, unique data for each woman for assessment of all sources of vitamin D, including sunlight exposure and various types of vitamin D supplementation, and their serum concentrations of 25 (OH) D was collected. The major finding was that lower serum 25(OH)D concentration was significantly associated with an increased risk of breast cancer supporting the hypothesis that vitamin D may play a protective role against breast cancer.

The median concentration of serum vitamin D in our study among cases was 15.3 (SD 15.6) ng/ml and among controls 16.7 (SD 20.1) ng/ ml. This was on average somewhat lower than the median concentration of serum vitamin D (25(OH)D) of 18.8 ng/ ml reported in a population based study of Pakistani population in Karachi in 2011 [61]. However, it is slightly higher than the reported median serum 25(OH)D concentration of 13.5 ng/ml in a clinical laboratory audit of Karachi Pakistan in the same year of 2011 [64]. In spite of the low latitude of Karachi and regular monthly sunshine throughout the year, VDD was found in 60.2% of the study participants with severe VDD (<12ng/ml) in 34.8%. of Pakistani women. Similar findings were reported in neighboring countries of India and China with high prevalence of VDD. In a study in Saudi Arabia, mean serum 25(OH)D concentrations were 13.1ng/ml [68]. This compares to concentrations in the United States western population where VDD was reported to be 28.9%. In the US study the incidence of VDD varied in different ethnic groups being highest among blacks (82.1%), followed by Hispanics (69.2%). Being a Muslim community, Pakistani women cover most of their body for cultural and religious reasons in a similar manner to Arab women who also have reduced concentrations of vitamin D. However, 54.5% of women in Singapore also had vitamin D deficiency, although they do not wear outer cloak and scarf like Muslim women. This may suggest Asian women have a predisposition to low concentrations of vitamin D. Possible other factors reducing sun exposure include sun avoidance behavior when outdoors, air pollution and dark skin pigmentation.

The study finding of association of lower serum 25(OH)D concentration with breast cancer is consistent with the results of a prospective observational mediterranean study, where deficient 25(OH)D concentrations were associated with node-positive high grade breast cancer [69]. Another recent pooled cohort showed that serum 25(OH)D concentration > 60 ng/ml lowered the risk of breast cancer by 80% compared to women with serum vitamin D <20ng/ml [70]. A case control study in China and meta-analysis of 21 independent studies also suggest that vitamin D may have a chemo-preventive effect against breast cancer [71]. A study among Caucasian population in UK also reported that low concentrations of serum 25(OH)D i.e. less than 20 ng/ml may increase risk of breast cancer [72]. However, in a Nurses’ Health Study cohort, high concentrations of serum vitamin D was associated with non- significant reduced risk of breast cancer in older women [72]. A population-based case-control study among premenopausal women of 289 cases and 595 matched controls, showed a significant inverse association between breast cancer risk and serum 25(OH)D. Compared with <30 nmol/L of vitamin D the ORs (95% CI) for the upper categories of 30–45, 45–60, ≥60 nmol/L were 0.68 (0.43–1.07), 0.59 (0.37–0.94) and 0.45 (0.29–0.70), respectively [73]. In a recent study among Spanish women, there were decreasing ORs of breast cancer with increasing concentration of serum vitamin D (OR per 10nmol/L = 0.88; 95%CI = 0.82–0.94) [74]. In a study of Indian women, low serum 25(OH)D concentrations (<20ng/ml) were associated with an increased risk of breast cancer [75]. In a previously published smaller study of Pakistani women, there was a similar association of VDD with increased risk of breast cancer [76]. Similarly study findings by Kim Y et al showed association of high vitamin D status with low breast cancer risk but strongly associated with better breast cancer survival [77].

This study results also show the association of vitamin D supplementation with both increased serum concentrations of vitamin D and significantly decreased breast cancer risk. A French Cohort study showed that current use (not past use) of calcium and Vitamin D supplements was associated with a lower risk of developing breast cancer in postmenopausal women, but not in pre-menopausal [78]. In a population-based case-control study, vitamin D supplement intake > 400 IU/d compared with no supplement intake was found to be independently associated with 24% reduced breast cancer risk (OR = 0.76, 95% CI = 0.59–0.98) while another 5 year follow up Sister Study of 50,884 participants showed that 25(OH)D concentrations were associated with a 21% reduced hazard of breast cancer and 11% reduced hazard of breast cancer with self-reported vitamin D supplementation [79, 80]. The Iowa Women’s Health Study has reported a small decrease in risk of breast cancer with vitamin D intake of >800 IU/d among postmenopausal women only. The protective effect of Vitamin D was highest in the first 5 years after baseline assessment of total intake but this protective effect decreased over time [81]. Three other case control studies by Rollison et al, Rossi et al and Crew et al also supported the protective effects of vitamin D supplements [82–84]. However, some studies demonstrate no protective association between vitamin D status and breast cancer risk and the association remains unclear [85].

Another cohort study showed that vitamin D was associated with lower incidence of cardiovascular disease, respiratory disease, and fractures but not total incident cancers [86].

According to a review by Feldman et al, in spite of inconsistent epidemiological and clinical trials results, some clinical studies agree with the beneficial effect of vitamin D in preventing cancer and that correcting VDD and usage of vitamin D supplements might have a role to reduce cancer incidence and improve cancer prognosis and outcome [87].

Vitamin D has been reported to have anticarcinogenic properties by stimulating apoptosis, inhibiting and slowing cell growth factors and improving cell cycle regulatory factors [88]. Further, 1,25(OH)_2_D may inhibit angiogenesis and down-regulate estrogen receptors [89]. Activated Vitamin D may exert its antitumor effects through the vitamin D receptor (VDR) that can modify the expression of target genes such as c-fos, p21, p27, and c-myc [90]. Another biological plausible mechanism of vitamin D is through its important role in both innate and adaptive immune system [91, 92]. Vitamin D functions as a transcription factor through the VDR, and has been shown to play an important role in mammary gland development and function, and is shown to be necessary and sufficient for tumor suppression in *in vitro* experiments and *in vivo* models of mammary tumor cells extracted from mice [93, 94]. Also a review suggests activation or restoration of the vitamin D regulated pathways has a potential to serve as avenue for human breast cancer prevention [88].

In this study, there was no association of total sun exposure score (a proxy measure of 25(OH)D concentrations) with serum 25(OH)D concentrations or breast cancer risk. This is contrary to those studies, particularly conducted at higher latitudes, which have shown a strong positive association between sunlight exposure concentrations and 25(OH)D concentrations and negative association with various cancers including breast [17]. Similarly, among a white population living in low latitude regions a multi-ethnic cohort nested case-control study, showed high concentrations of 25(OH)D were associated with a reduced risk of postmenopausal breast cancer. In a study of data from the Ontario Cancer registry, breast cancer risks were reduced among women who had increasing sun exposure at earlier life (ages 10–19 years) These studies emphasize the importance of natural sources of vitamin D though sun exposure. The absence of an association between total sun exposure score and vitamin D and breast cancer in our study could also be due to inaccuracies in the collection of this type of data, in particular the recall of past sun exposure time. Serum 25(OH)D is a useful biomarker for measuring an individual’s recent exposure to sun exposure but may not correlate with lifetime sun exposure in different seasons. Moreover, it should be noted that Karachi is the fourth most polluted city in the world with the presence of heavy smog and air pollution associated with rapid industrialization. This further has the potential to affect access to sun exposure and its UV-B light, causing decreased penetration of ultraviolet B radiation from the sun [95–97].

This study showed that there was no association of vitamin D with tumor characteristics, ER or PR status or stage of breast cancer which is consistent with similar findings in a Chinese population [98]. It is in contrast with research finding of an association of high 25(OH)D concentrations with lower tumor size at early breast cancer diagnosis [99] or ER/PR status of tumor [100].

Some limitations of the study are to be noted. First, measurement of serum 25(OH)D concentrations was done by a single venous blood sample collected at one point in time which may not be necessarily associated with 25(OH)D concentrations at the time of breast cancer initiation or progression. However, findings from an important clinical trial suggest that serum 25(OH)D concentration at a single time point may be representative of long-term vitamin D status over a five-year period [70]. Another study showed that the correlation coefficient for measurement of serum 25(OH)D concentrations in samples collected in 1994 and 2008 ranged from 0.42 to 0.52, and was 0.80 when measured a year apart [101]. Therefore, single measurement of serum 25(OH)D was based on the findings that an individual’s serum 25(OH)D concentration remain relatively stable over time [102]. Moreover, this type of misclassification would be non-differential to both cases and controls. Second, bias of reverse causation is inherent in case control studies, for example, due to the timing of blood collection for assay of 25(OH)D concentrations after breast cancer diagnosis, the presence of breast cancer cells and catalytic enzymes may affect the assays. Survival studies show that breast cancer itself can cause lower serum concentrations of 25(OH)D [103] but in this study only newly diagnosed incident cases of breast cancer were enrolled. Even though participants were newly diagnosed with breast cancer in the study, they may very well have had the disease for quite some time, since national breast cancer screening is not available in Pakistan and awareness about symptoms of breast cancer is low. Third, it was a hospital-based study with lack of generalizability and Berkson’s bias [104] make it difficult to generalize as people seen in any hospital-based case-control study are different in their clinical states from general population. However, all efforts were made done to avoid selection bias by proper selection of cases and controls with precise case definition and exposure definition, selection of controls from general practice (independent of the exposure status), and uniform data collection procedures. Lastly, 1,25(OH)_2_D concentrations may be more relevant due to the enzymatic conversion of 25(OH)D to 1,25(OH)_2_D, but it was not available in our study. Despite some limitations, the strengths of this study are considerable, including a large sample size with good statistical power, a high response rate and good participation in both cases and controls, best methods of measurement of all the variables using validated questionnaires and excellent laboratory methods for ELISA assay, standardized, sample storage at -80C, blind assessment of laboratory personal testing for 25 OH(D), which ensured comparability of information in both cases and controls, were the strengths of this study. Lastly, measurement of vitamin D from all sources was assessed especially serum concentration of 25(OH)D measurement was carried out which is the gold standard for assessing vitamin D status reflecting integration of cutaneous synthesis, as well as dietary and supplemental vitamin D intake.

Overall this study suggests that there may be a potential role of cost- effective vitamin D in reducing breast cancer incidence in a developing and limited resources country like Pakistan, in contrast to more affluent societies. Pakistan has overburdened cancer care services in the health and development systems with an inefficient palliative care which largely remains unknown and neglected area. Cancer treatment facilities are situated in big cities and are beyond the reach of the majority of the population. Pakistan’s ratio of health services to population is one of the highest (1:90 million) [105].

## Conclusion

To conclude, in this study, we observed that lower serum 25(OH)D concentration was associated with a higher risk of breast cancer supporting the hypothesis that deficient concentrations of serum vitamin D may contribute to the process of carcinogenesis and its correction may play a role in protection against breast cancer. VDD may be a modifiable risk factor for breast cancer and restoring adequate concentrations of vitamin D may be a safe and affordable approach that could reduce the incidence of VDD and breast cancer. This finding is also supported by the results showing that vitamin D supplementation in Pakistani women was associated with both increased concentrations of vitamin D and reduced incidence of breast cancer. However, further studies, for example a prospective cohort study to confirm our findings in a large prospective study for temporality, or large randomized clinical trials, are needed to draw firmer conclusions and clarify this association in a dose-response relationship. Such large RCTs may confirm the optimal concentrations of serum 25(OH)D for breast cancer prevention, which may be effective and low-cost strategy for women to reduce their risk of breast cancer through preventing VDD and insufficiency.

In Pakistani women raising and maintaining serum 25(OH)D concentration at the population concentration can be achieved through public awareness campaigns to improve 25(OH)D concentrations through lifestyle changes that promote sensible sun exposure, consumption of foods that contain vitamin D and are fortified with vitamin D, and use of vitamin D supplements.

## Supporting information

S1 Data Data underlying the presented results. (SAV)

S1 Table Weights given to sun exposure variables. (DOCX)

S2 Table Serum 25(OH)D concentration and intake of Vitamin D supplements among breast cancer cases & controls. (DOCX)

S3 Table Distribution of sun exposure variables among the breast cancer cases and controls. (DOCX)
