# Supplementary material for: A multicenter case control study of association of vitamin D with breast cancer among women in Karachi, Pakistan
Source: PLoS One. 2020 Jan 22;15(1):e0225402. doi: 10.1371/journal.pone.0225402 (PMC6975526; doi:10.1371/journal.pone.0225402)
Supplement: S1 Table — (DOCX) [file pone.0225402.s002.docx]

**Supporting information**

**S1 Table.** **Weights given to sun exposure variables**

| **Variable/item** | **Weights given** |
| --- | --- |
| Part of the body exposed based on attire used outside | 1 if exposed (100% UVB absorption)  0 if covered (0 UVB absorption)  0.5 if partially covered (50% UVB absorption) |
| Use of sunscreen on different parts of body | 1 if no use (100% UVB absorption)  0.08 if sunscreen (8% UVB absorption) |
| Sun avoidance behavior | 1 if no protection practices (100% UVB absorption)  0.4 if seeking shade under trees/building etc  (40% UVB absorption) |
| Weather outdoors | 1 if sunny (100% UVB absorption)  0.5 if cloudy (50% UVB absorption)  0.75 if sunny/cloudy(75% UVB absorption) |
| Skin tone | 0.80 if Type 1 (80% UVB absorption)  0.675 if Type II (67.5% UVB absorption)  0.55 if Type III (55% UVB absorption)  0.425 if Type IV (42.5% UVB absorption)  0.30 if Type V (30% UVB absorption) |
