# Supplementary material for: A multicenter case control study of association of vitamin D with breast cancer among women in Karachi, Pakistan
Source: PLoS One. 2020 Jan 22;15(1):e0225402. doi: 10.1371/journal.pone.0225402 (PMC6975526; doi:10.1371/journal.pone.0225402)
Supplement: S2 Table — (DOCX) [file pone.0225402.s003.docx]

**S2 Table. Vitamin D level and intake of vitamin D supplements among breast cancer cases & controls**

| **Serum Vitamin D ng/ml** | | | **Case** | | **Control** | | **p value*** |
| --- | --- | --- | --- | --- | --- | --- | --- |
| **Vitamin D deficiency (<20)** | **Vitamin D supplements** |  | **n** | **%** | **n** | **%** | 0.001 |
|  |  | nonuser | 139 | 72.0% | 176 | 50.6% |  |
|  |  | user | 54 | 28.0% | 172 | 49.4% |  |
| **Vitamin D insufficiency (20-30)** | **Vitamin D supplements** |  |  |  |  |  | 0.001 |
|  |  | nonuser | 25 | 59.5% | 31 | 27.2% |  |
|  |  | user | 17 | 40.5% | 83 | 72.8% |  |
| **Vitamin D sufficiency (>30)** | **Vitamin D supplements** |  |  |  |  |  | 0.001 |
|  |  | nonuser | 22 | 44.9% | 31 | 20.4% |  |
|  |  | user | 27 | 55.1% | 121 | 79.6% |  |
