# Supplementary material for: A multicenter case control study of association of vitamin D with breast cancer among women in Karachi, Pakistan
Source: PLoS One. 2020 Jan 22;15(1):e0225402. doi: 10.1371/journal.pone.0225402 (PMC6975526; doi:10.1371/journal.pone.0225402)
Supplement: S3 Table — (DOCX) [file pone.0225402.s004.docx]

**S3 Table. Distribution of sun exposure variables among the breast cancer cases and controls in the multicenter case control study**

|  |  | **case** | | | | **control** | | | |
| --- | --- | --- | --- | --- | --- | --- | --- | --- | --- |
|  | **Sun exposure variables** | **Mean** | **Median** | **Range** | **SD*** | **Mean** | **Median** | **Range** | **SD*** |
| IA | **Total sun exposure score** |  |  |  |  |  |  |  |  |
|  | Score of Sun exposure in Summer per week | 36.5 | 3.6 | 689.0 | 79.1 | 32.2 | 1.6 | 1545.0 | 99.6 |
|  | Score of Sun exposure in Winter per week | 56.4 | 19.4 | 716.3 | 97.6 | 48.0 | 11.5 | 1545.0 | 117.6 |
| IB | **Components of sun exposure score** |  |  |  |  |  |  |  |  |
|  | Sun exposure in summer(days/week) | 2.7 | 1 | 7 | 2.9 | 2.6 | 1 | 7 | 2.9 |
|  | Sun exposure in winters(days/week) | 3.6 | 3 | 7 | 3 | 3.3 | 3 | 7 | 3 |
|  | Sun exposure in summers (minutes/ day) | 20.7 | 7 | 260 | 37.5 | 18 | 5 | 600 | 44.5 |
|  | Sun exposure in winters (minutes/day) | 29.8 | 15 | 300 | 44.1 | 25 | 10 | 600 | 51.5 |
|  | Sun Exposure during summer in minutes/ week | 108.8 | 20 | 1820 | 233.7 | 93.6 | 10 | 4200 | 281.7 |
|  | Sun Exposure during winter in minutes/ week | 166.2 | 60 | 2100 | 290.1 | 137.8 | 35 | 4200 | 328.5 |
